# Possible Cause of Liver Failure in Patient with Dengue Shock Syndrome

**DOI:** 10.3201/eid1907.121820

**Published:** 2013-07

**Authors:** Apichai Khongphatthanayothin, Atchara Mahayosnond, Yong Poovorawan

**Affiliations:** Bangkok Hospital Medical Center, Bangkok, Thailand (A. Khongphatthanayothin);; Chulalongkorn University, Bangkok (A. Khongphatthanayothin, A. Mahayosnond, Y Poovorawan)

**Keywords:** dengue hemorrhagic fever, dengue shock syndrome, liver failure, portal blood flow, sinusoidal obstruction, viruses, dengue virus, dengue, DENV

**To the Editor:** We report a rare hepatic ultrasonograph finding for a patient with liver failure associated with dengue virus (DENV) infection. This finding might shed light on the pathogenesis of liver involvement in this disease.

In March 2006, a 10-year-old previously healthy boy was hospitalized for a 3-day history of fever, headache, and nausea/vomiting. Fever subsided on the day of admission, but the patient was in shock (blood pressure 80/40 mm Hg) and had gastrointestinal bleeding and hematuria. Physical examination showed an obese, confused patient with generalized petechiae and hepatomegaly. The initial diagnosis was dengue shock syndrome (DSS). The patient was intubated and received intravenous fluid infusion, packed red blood cells, ceftriaxone, sodium bicarbonate, and ranitidine before being transferred to King Chulalongkorn Memorial Hospital in Bangkok. The patient’s blood pressure increased to 130/90 mm Hg after the initial fluid resuscitation (28 mL/kg free flow), and systolic pressure remained at ≈130 mm Hg until transfer.

Laboratory examinations found 14,930 leukocytes/mm^3^, hemoglobin 16.4 g/dL, hematocrit 48.2%, platelet 18,000/mm^3^, blood urea nitrogen 33 mg/dL, creatinine 1 mg/dL, sodium 128 mEq/L, potassium 6.2 mEq/L, chloride 91 mEq/L, total CO_2_ 5 mEq/L, total bilirubin 6.9 mg/dL, direct bilirubin 3.9 mg/dL, aspartate transaminase 3,507 IU/L, alanine transaminase 2,775 IU/L, prothrombin time 43 seconds (international normalized ratio 3.4), and partial thromboplastin time 93.5 s (control 28.7 s). Blood and urine cultures showed negative results. Serum was positive for IgM against DENV. Unfortunately, we did not investigate other viral causes of liver failure. 

DSS with liver failure was diagnosed and treated with intravenous fluid, sodium bicarbonate, omeprazole, fresh frozen plasma, platelet transfusion, vitamin K, and recombinant factor VIIa concentrate (NovoSeven; Novo Nordisk, Bagsvaerd, Denmark). Despite stable blood pressure over the next 6 days, liver enzymes continued to rise with progressive jaundice (Technical Appendix). Hepatic ultrasonograph on the second day after admission showed totally reversed direction of portal venous blood flow away from the liver (Figure, panel A), becoming bidirectional on the following day and, finally, reverting to normal direction (although with low velocity) 3 days later (Figure, panel B). Despite improved hemodynamic status, progressive encephalopathy and gastrointestinal bleeding developed and were unresponsive to treatment. Six days later, the patient died of pulmonary hemorrhage and progressive respiratory failure.

**Figure:**
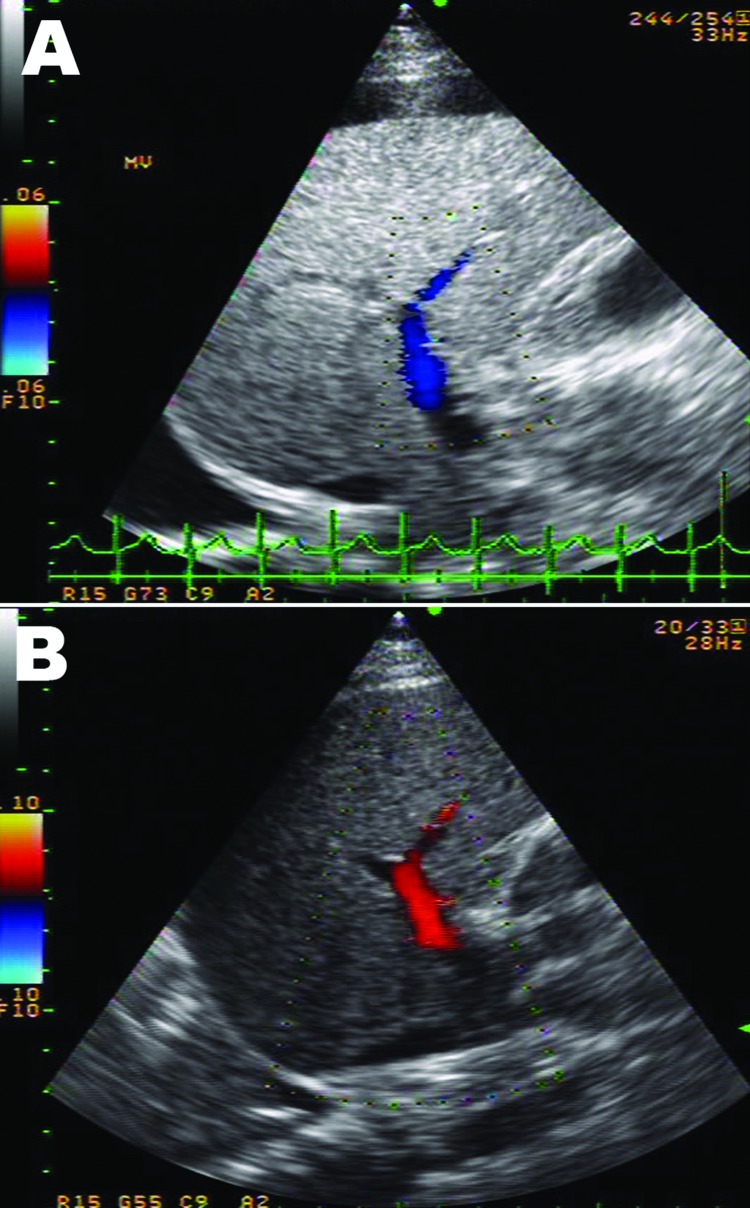
Ultrasonograph with Doppler image of the liver of a 10-year-old boy with liver failure associated with dengue virus infection. A) Day 2 of hospitalization, showing reversed direction of blood flow in the right branch of the portal vein (hepatofugal flow). There was diffuse increased liver parenchymal echo, swelling of the gallbladder wall, and right pleural effusion. B) Day 5 of hospitalization, showing returning normal direction of portal venous flow (hepatopetal flow). Liver parenchymal echo changed to normal. Pleural fluid and swelling of the gallbladder wall also disappeared.

DENV infection is one of the most prevalent emerging infectious diseases affecting children and one of the leading causes of liver failure in tropical countries (*1*,*2*). Although liver involvement in patients with dengue hemorrhagic fever is well known, the mechanism for DENV-induced liver injury is still a mystery. Liver autopsy specimens of terminal DSS patients generally showed massive or focal necrosis with little or no recruitment of polymorphonuclear cells or lymphocytes (*3*,*4*). Ultrasonograph images from patients with liver failure caused by acetaminophen poisoning or hepatitis B indicate increased portal vein flow and normal flow velocity to the damaged liver (*5*). Decreased portal vein flow velocity and reversal of the flow direction is seen in the terminal stage of hepatic cirrhosis and a few other conditions such as hepatic sinusoidal obstruction (hepatic veno-occlusive disease), arterioportal fistula, extrahepatic portal vein thrombosis, and hepatic venous outflow obstruction (*6*). This finding is unusual in other instances of toxin- or virus-induced liver failure and might contribute to the understanding of the mechanism of liver involvement in patients with DENV infection.

We previously reported increased portal vein congestion during the toxic stage of DENV infection (*7*). At defervescence, the portal vein was dilated and blood flow velocity was decreased. This finding is usually observed for patients with high resistance in the hepatic sinusoidal capillary network, such as those with liver cirrhosis, and is correlated with the degree of portal venous hypertension (*8*). We postulate that DENV infection of the liver might affect the sinusoidal endothelial or Kupffer cells in a way that causes obstruction to the hepatic sinusoidal capillary lumen resulting in decreased portal venous blood velocity and flow to the liver and, when severe, shunting of portal blood away from the liver (hepatofugal flow). Because portal venous blood comprises 75% of total hepatic blood (*6*), this condition coupled with decreased hepatic arterial blood flow as a consequence of shock might have led to severe and irreversible liver damage in this patient. This hypothesis can be further supported by a pathology study of the skin in patients with DENV infection, which showed endothelial swelling and extrusion of its plasma membrane into the capillary lumen, resulting in narrowing of the capillary lumen (*9*). Of note are the similarities between clinical findings in patients with DENV infection and sinusoidal obstruction syndrome such as hepatomegaly, ascites, right pleural effusion, swelling of the gall bladder wall, and decreased velocity or reversed direction of portal blood flow (*10*).

In conclusion, we report a case of liver failure from DENV infection with reversal of portal venous blood flow. We postulate that hepatic sinusoidal obstruction coupled with shock might be the underlying mechanism of liver failure in this disease.

Technical Appendix Clinical and laboratory data for patient with liver failure associated with dengue virus infection.
