# Supplementary material for: Possible Cause of Liver Failure in Patient with Dengue Shock Syndrome
Source: Emerg Infect Dis. 2013 Jul;19(7):1161–3. doi: 10.3201/eid1907.121820 (PMC3713982; doi:10.3201/eid1907.121820)
Supplement: Technical Appendix — Clinical and laboratory data for patient with liver failure associated with dengue virus infection. [file 12-1820-Techapp-s1.pdf]

Article DOI: http://dx.doi.org/10.3201/eid1907.121820

Possible Cause of Liver Failure in Patient with Dengue Shock Syndrome

Technical Appendix

Technical Appendix Table. Serial clinical and laboratory data for patient with liver failure and dengue shock syndrome.

	Day after onset of fever							
Parameter	3–4	4–5	5–6†	6–7	7–8	8–9†	9–10	10–11
Heart rate	101–130	100	90	100	100	100	110	100→0
BP, mm Hg‡								
Systolic	80–155	81–151	127–150	122–138	115–130	111–127	106–145	99→0
Diastolic	40–93	52–96	65–90	66–92	57–66	45–60	36–68	32→0
Hematocrit	48.2	24.6	30.7	33.6	27.8	26.9	28.7	24.1
ALT	2,775	4,490	4,720	3,098	2,011	1,729	1,725	543
AST	3,507	11,660	>7,000	8,440	4,082	3,099	2,600	968
Bilirubin								
Total	6.9	9.7	12.2	21.6	24.1	34.8	32.1	23.8
Direct	3.9	5.5	6.3	12.1	14.2	18.4	6.0	14.1
PT, INR	3.4	2.4	2.4	NA	2.3	2.3	2.1	NA

*BP, blood pressure; ALT, alanine transaminase; AST, aspartate transaminase; PT, prothrombin time; INR, international normalized ratio); NA, not available.
†Dates when liver ultrasonography (Figure) was performed.
‡1 episode of BP 81–85/52–54 mm Hg occurred for 15 minutes, 12 hours after hospital admission; otherwise, the lowest systolic BP was 108 mm Hg.
